# Metformin Induces Apoptosis in Human Pancreatic Cancer (PC) Cells Accompanied by Changes in the Levels of Histone Acetyltransferases (Particularly, p300/CBP-Associated Factor (PCAF) Protein Levels)

**DOI:** 10.3390/ph16010115

**Published:** 2023-01-12

**Authors:** Izabela Szymczak-Pajor, Józef Drzewoski, Ewa Świderska, Justyna Strycharz, Anna Gabryanczyk, Jacek Kasznicki, Marta Bogdańska, Agnieszka Śliwińska

**Affiliations:** 1 Department of Nucleic Acid Biochemistry, Medical University of Lodz, 251 Pomorska Str., 92-213 Lodz, Poland; 2 Central Teaching Hospital of the Medical University of Lodz, 251 Pomorska Str., 92-213 Lodz, Poland; 3 Department of Medical Biochemistry, Medical University of Lodz, 6/8 Mazowiecka Str., 92-215 Lodz, Poland; 4 Department of Internal Diseases, Diabetology and Clinical Pharmacology, Medical University of Lodz, 251 Pomorska Str., 92-213 Lodz, Poland; 5 Student Scientific Society of Civilization Diseases, Medical University of Lodz, 251 Pomorska Str., 92-213 Lodz, Poland

**Keywords:** metformin, pancreatic cancer (PC), histone acetyltransferases (HATs), histone deacetylases (HDACs)

## Abstract

Accumulating evidence (mainly from experimental research) suggests that metformin possesses anticancer properties through the induction of apoptosis and inhibition of the growth and proliferation of cancer cells. However, its effect on the enzymes responsible for histone acetylation status, which plays a key role in carcinogenesis, remains unclear. Therefore, the aim of our study was to evaluate the impact of metformin on histone acetyltransferases (HATs) (i.e., p300/CBP-associated factor (PCAF), p300, and CBP) and on histone deacetylases (HDACs) (i.e., SIRT-1 in human pancreatic cancer (PC) cell lines, 1.2B4, and PANC-1). The cells were exposed to metformin, an HAT inhibitor (HATi), or a combination of an HATi with metformin for 24, 48, or 72 h. Cell viability was determined using an MTT assay, and the percentage of early apoptotic cells was determined with an Annexin V-Cy3 Apoptosis Detection Assay Kit. Caspase-9 activity was also assessed. *SIRT-1*, *PCAF*, *p300*, and *CBP* expression were determined at the mRNA and protein levels using RT-PCR and Western blotting methods, respectively. Our results reveal an increase in caspase-9 in response to the metformin, indicating that it induced the apoptotic death of both 1.2B4 and PANC-1 cells. The number of cells in early apoptosis and the activity of caspase-9 decreased when treated with an HATi alone or a combination of an HATi with metformin, as compared to metformin alone. Moreover, metformin, an HATi, and a combination of an HATi with metformin also modified the mRNA expression of *SIRT-1*, *PCAF*, *CBP*, and *p300*. However, metformin did not change the expression of the studied genes in 1.2B4 cells. The results of the Western blot analysis showed that metformin diminished the protein expression of PCAF in both the 1.2B4 and PANC-1 cells. Hence, it appears possible that PCAF may be involved in the metformin-mediated apoptosis of PC cells.

## 1. Introduction

Metformin, which is a derivative of biguanide, is still the most commonly prescribed oral glucose-lowering medication for the treatment of type 2 diabetes mellitus (T2DM). Its anti-hyperglycemic action results, primarily, from the inhibition of hepatic gluconeogenesis, which leads to a reduction in hepatic glucose output. The drug also enhances the insulin sensitivity of the liver and switches hepatocytes from an anabolic pathway (i.e., gluconeogenesis) to a catabolic pathway (i.e., glycolysis), which consumes less energy. Metformin also increases insulin sensitivity in the peripheral tissues, which results in increased glucose uptake and consumption by skeletal muscle and adipose tissue [1]. Interestingly, a growing number of clinical studies suggest that, in addition to controlling glucose metabolism in people with chronic hyperglycemia, metformin may also play a role in cancer prevention and therapy [2]. Nearly two decades ago, a case–control study of cancer incidence in patients with newly diagnosed T2DM who had or had not received metformin found metformin treatment to reduce the risk of cancer in patients [3]. Later, a meta-analyses indicated that metformin treatment bestowed preventive effects, reduced all-cause mortality, and led to improvements in overall survival outcomes in patients with breast, liver, colorectal, and pancreatic cancers (PCs) [4,5,6,7]. 

The underlying mechanism of the antitumor properties of metformin remain only partially understood. It may exert an indirect effect through its glucose-lowering action, causing reductions in hyperglycemia, insulin resistance, and insulin-like growth factor-1 (IGF-1), as well as exerting a direct effect through various mechanisms. Firstly, it has been proven that metformin suppresses the activity of mitochondrial glycerol-3-phosphate dehydrogenase (mG3PDH), thereby elevating the NADH/NAD^+^ ratio and decreasing the conversion of pyruvate to lactate, which leads to the accumulation of lactate and a decrease in hepatic gluconeogenesis [8]. Secondly, metformin inhibits the activity of mitochondrial electron transport chain (ETC) complex I, thereby triggering a decrease in ATP production and an increase in the [AMP]_i_/[ATP]_i_ and [ADP]_i_/[ATP]_i_ ratios [8,9]. In conditions of excess AMP, AMP binds to the γ-subunit of AMPK, which induces αβγ-heterotetrameric complex formation and stimulates its binding with liver kinase B1 (LKB1). LKB1 is a tumor suppressor that phosphorylates the AMPKα subunit in the Thr^172^ position, thereby activating AMPK; this stimulates numerous cellular events, including a reduction in inflammatory factor activity (i.e., NF-ĸB), an elevation in protein acetylation, a decrease in endoplasmic reticulum stress and the production of reactive oxygen species (ROS), and a reduction in the insulin/IGF-1-induced stimulation of mTORC1/2 complexes. AMPK and mTORC1 are known as two key metabolic regulators. mTOR is a key mediator in the PI3K/Akt (phosphatidylinositol-3-kinase/Akt) signaling pathway, which is the most frequently deregulated pathway in human cancer. As a result of AMPK activation, metformin negatively affects the PTEN/PI3K/Akt/mTOR signaling pathway, which leads to disturbances in cell growth and shortens survival [10,11,12]. Metformin-activated AMPK inhibits mTOR, which leads to the reduced phosphorylation of its target proteins such as initiation factor binding E4 (4EBPs) and ribosomal S6 kinase (S6Ks), thereby resulting in the inhibition of protein synthesis and the cell cycle and suppression of angiogenesis and cell proliferation [13,14].

Additionally, metformin has demonstrated systemic effects associated with the suppression of tumor development via the attenuation of insulin/insulin-like growth factor (IGF)-1 signaling, such as decreasing proinflammatory cytokine levels, reducing the expression of cell adhesion molecules, inhibiting the Warburg effect, and causing lactate secretion by tumors [15,16]. Furthermore, it was reported that the direct action of metformin contributed to a decreased expression of transcription factors, namely, specificity proteins such as Sp1, Sp3, and Sp4, as well as pro-oncogenic Sp-regulated genes [17].

It is known that the level of chromatin condensation is highly dynamic. Post-translational modifications of histone and nonhistone proteins may be rapidly removed or added. Histone acetylation, which occurs extensively in the histone tail and occasionally in the core, is based on the addition of an acetyl moiety to the ϵ-amino terminal of lysine residues in the tail domain of the core histone (mostly in H3 and H4). As a result, the positive charge on the histone is neutralized, lessening the electrostatic interaction between DNA and histones and resulting in the formation of relaxed, active chromatin [18].

In contrast, the removal of acetyl groups from the acetylated histones, known as histone deacetylation, triggers the formation of condensed and inactive chromatin [19]. Histone acetylation status is maintained by two classes of enzymes: histone acetyltransferases (HATs), i.e., p300/CBP-associated factor (PCAF), p300, and CBP, and deacetylases (HDACs), i.e., SIRT-1. Imbalances between acetylation and deacetylation status or the aberrant enzymatic activity of HDACs and HATs are frequently observed in various types of cancers including PC [20,21]. Notably, altered HDAC activity can lead to the deregulation of crucial biological processes such as apoptosis, cell proliferation, differentiation, and migration [20].

Epigenetic regulators act as activators of gene expression by initiating the opening of chromatin, giving transcriptional factors access to certain genes in the genome. In cells, PCAF and CBP/p300 have been identified in the same complexes; however, their patterns of substrate specificity are different. CBP/p300 is responsible for the acetylation of lysines on all four histones, whereas PCAF acetylates lysine 14 on histone H3 [22,23]. SIRT-1 is an NAD^+^-dependent class III HDAC that plays a key role in the regulation of numerous biological processes including metabolism, aging, oncogenesis, and cancer progression [24,25].

The present study evaluates whether an HDAC (SIRT-1) and HATs (PCAF, p300, and CBP) are involved in the metformin-evoked induction of apoptosis in PC cells.

## 2. Results

### 2.1. Metformin Decreased the Viability of 1.2B4 and PANC-1 Cells

**Figure 1:**
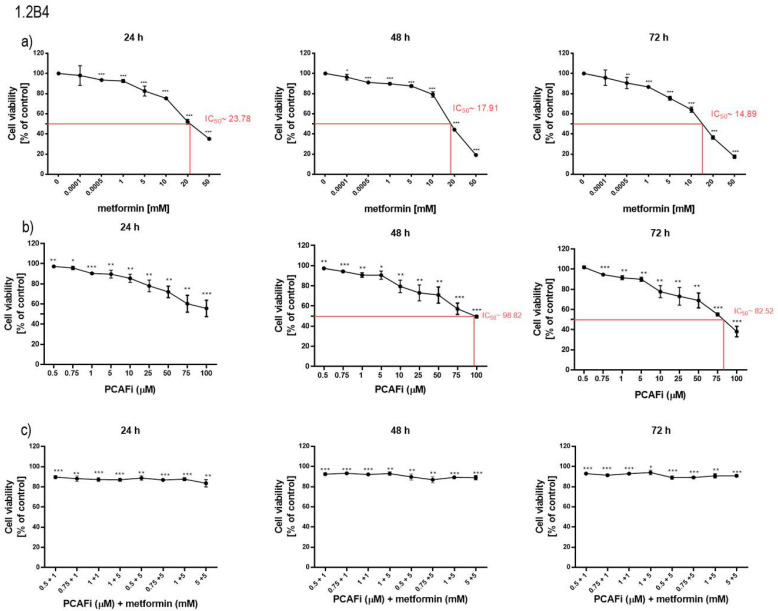

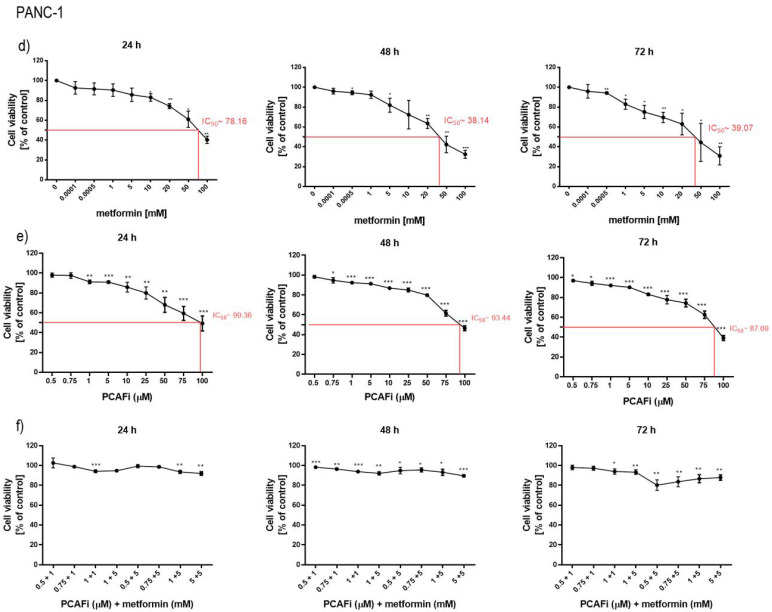
The effect of metformin, HAT inhibitor (HATi), and combination of HATi with metformin on viability of PC cells ((**a**) metformin; (**b**) HATi; (**c**) HATi + metformin in 1.2B4; (**d**) metformin; (**e**) HATi; (**f**) HATi + metformin in PANC-1). Cell viability was evaluated by MTT assay. The 1.2B4 and PANC-1 cells were treated with metformin (0.0001–100 mM), HATi (0.5–100 µM), and HATi (0.5, 0.75, 1, and 5 µM) with metformin (1 and 5 mM) for 24 h, 48 h, and 72 h. After completion of treatment, MTT was added for 4 h. Then, to dissolve formazan crystals, DMSO was added and absorbance at 570 nm was measured with a microplate reader. The data are presented as the mean ± SD of the results of three independent experiments. * *p* < 0.05, ** *p* < 0.01, and *** *p* < 0.001.

The effect of metformin (0.0001–100 mM) on the viability of PC cells was determined via an MTT assay (Figure 1a,d). Metformin significantly reduced 1.2B4 cell viability in a dose- and time-dependent manner (IC_50_ = 23.78 mM for 24 h vs. IC_50_ = 17.91 mM for 48 h vs. IC_50_ = 14.89 for 72 h). In addition, the exposure of 1.2B4 cells to high metformin concentrations induced a markedly greater decrease in cell viability after 48 and 72 h than after 24 h. In the case of PANC-1 cells, metformin exerted a dose- and time-dependent cytotoxic effect after 24, 48, and 72 h of incubation. However, similar cytotoxic potentials were observed between the 48- and 72-h exposures (IC_50_ = 38.14 mM for 48 h vs. IC_50_ = 39.07 mM for 72 h). These results suggest that after 48 h of exposure to ~40 mM of metformin, the cytotoxic effect seems to be independent of the incubation time in PANC-1 cells. The obtained results also suggest that 1.2B4 cells are more sensitive to metformin compared to PANC-1 cells.

### 2.2. Metformin Induces Apoptosis of 1.2B4 and PANC-1 Cells

**Figure 2:**
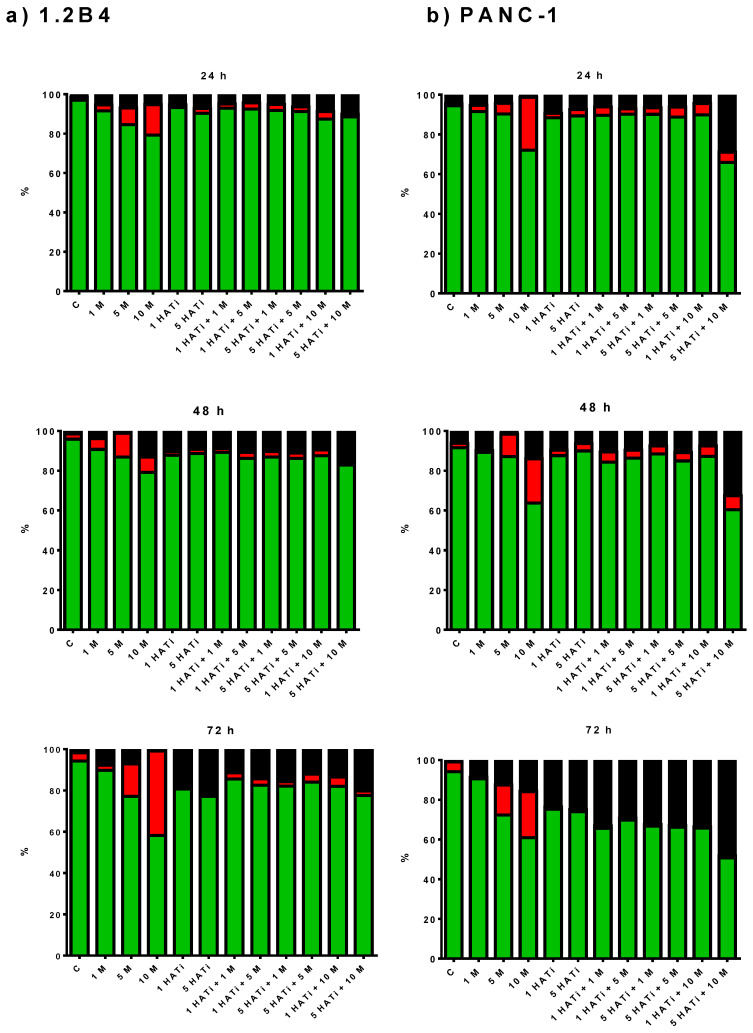
Proapoptotic action of metformin in PC cells. The 1.2B4 (**a**) and PANC-1 cells (**b**) were treated with metformin (1, 5, and 10 mM), HATi (1 and 5 µM), and combination of HATi (1 and 5 µM) with metformin (1, 5, and 10 mM) for 24, 48, and 72 h. Early apoptosis was assessed via combined staining with Ann-Cy3 and CFDA. Then, the samples were analyzed by flow cytometry within 1 h. Green bars—live cells; red bars—apoptotic cells; yellow bars—necrotic cells; and black bars—dead cells. Additional figures of the flow cytometry results have been included in Supplementary Files.

The effect of metformin on PC cell apoptosis was determined using an Annexin V-Cy3 Apoptosis Detection Assay Kit with flow cytometry. Based on the results obtained by the MTT assay, it was decided that a 1, 5, and 10 mM concentration of metformin would be used. The percentages of viable, early apoptotic, and dead 1.2B4 and PANC-1 cells are displayed in Figure 2a,b. Metformin induced the dose-dependent and time-dependent apoptosis of 1.2B4 and PANC-1 cells. It demonstrated pronounced proapoptotic action at doses of 5 mM and 10 mM. Only a small percentage of apoptotic cells were observed after treatment with 1 mM of metformin in both 1.2B4 and PANC-1 cells.

### 2.3. Metformin Increases Caspase-9 Activity

**Figure 3:**
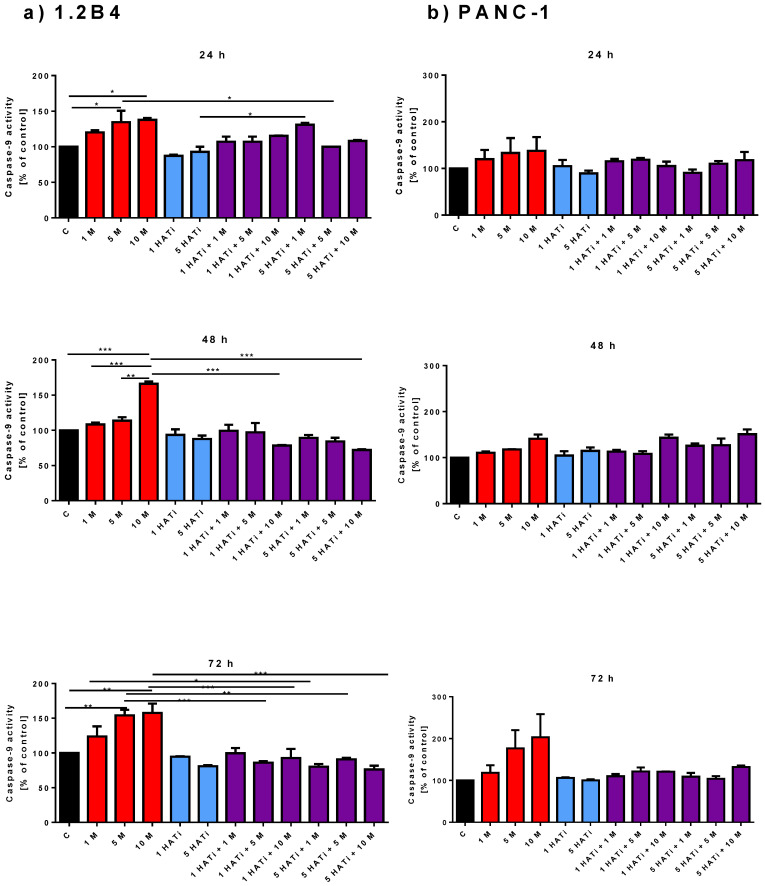
The effect of metformin, HATi, and combination of HATi with metformin on the activity of caspase-9 in 1.2B4 (**a**) and PANC-1 (**b**) cells. Activity of caspase-9 in 1.2B4 (**a**) and PANC-1 (**b**) cells was determined by Caspase-9 Colorimetric Activity Assay Kit. The cells were treated with metformin (1, 5, and 10 mM; red bars), HATi (1 and 5 µM; blue bars), and combination of HATi (1 and 5 µM) with metformin (1, 5, and 10 mM; violet bars) for 24, 48, and 72 h. Data are expressed as the mean ± SD. * *p* < 0.05, ** *p* < 0.01, and *** *p* < 0.001.

To confirm the results obtained by flow cytometry, the effect of metformin on caspase-9 activity was determined as a marker of apoptotic cell death (Figure 3a,b). Metformin was found to increase caspase-9 activity in both 1.2B4 and PANC-1 cells in a dose-dependent manner. A pronounced increase in caspase-9 activity in 1.2B4 cells was detected in response to 10 mM of metformin after 24, 48, and 72 h, and to 5 mM of metformin after 24 and 72 h.

### 2.4. Metformin Slightly Modifies mRNA Expression of SIRT-1, PCAF, p300, and CBP in PANC-1 Cells, but Not in 1.2B4 Cells

The effect of metformin on the mRNA expression of an HDAC (SIRT-1) and HATs (PCAF; p300; and CBP) was determined in 1.2B4 (Figure 3a) and PANC-1 (Figure 3b) cells by using a qRT-PCR. 

In the 1.2B4 cells, metformin had no statistically significant effect on the mRNA expression of SIRT-1, PCAF, CBP, and p300. However, exposure to 5 mM of metformin for 48 h and 1 mM and 5 mM of metformin for 72 h upregulated the SIRT-1 mRNA expression. No significant increase in PCAF mRNA expression was seen after the 24-, 48-, and 72-h treatment. CBP mRNA expression seemed to be slightly increased after 48 and 72 h of exposure to metformin. Moreover, no changes in p300 mRNA expression were found in response to exposure to metformin for 24, 48, and 72 h. Taken together, although some slight expression changes were seen after the metformin treatment, it appears that it did not significantly alter the expression of the studied genes in 1.2B4 cells.

In the PANC-1 cells, metformin appeared to have a dose-dependent effect on the expression of SIRT-1, PCAF, and CBP after the 24-h incubation. SIRT-1 mRNA was slightly increased after exposure to 1 mM of metformin for 72 h. Interestingly, after 48 and 72 h of treatment, no statistically significant change in PCAF and CBP mRNA was observed, although they demonstrated similar expression patterns. In addition, the p300 mRNA expression was unaffected by the metformin treatment.

### 2.5. Metformin Decreases Protein Expression of PCAF in Both 1.2B4 and PANC-1 Cells

**Figure 4:**
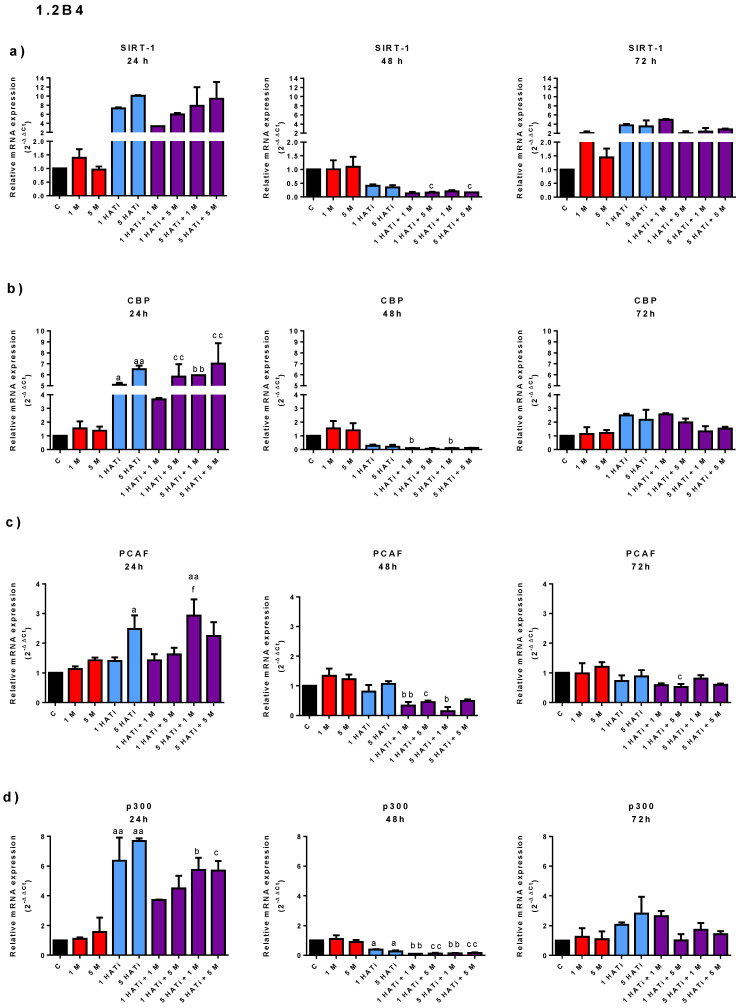

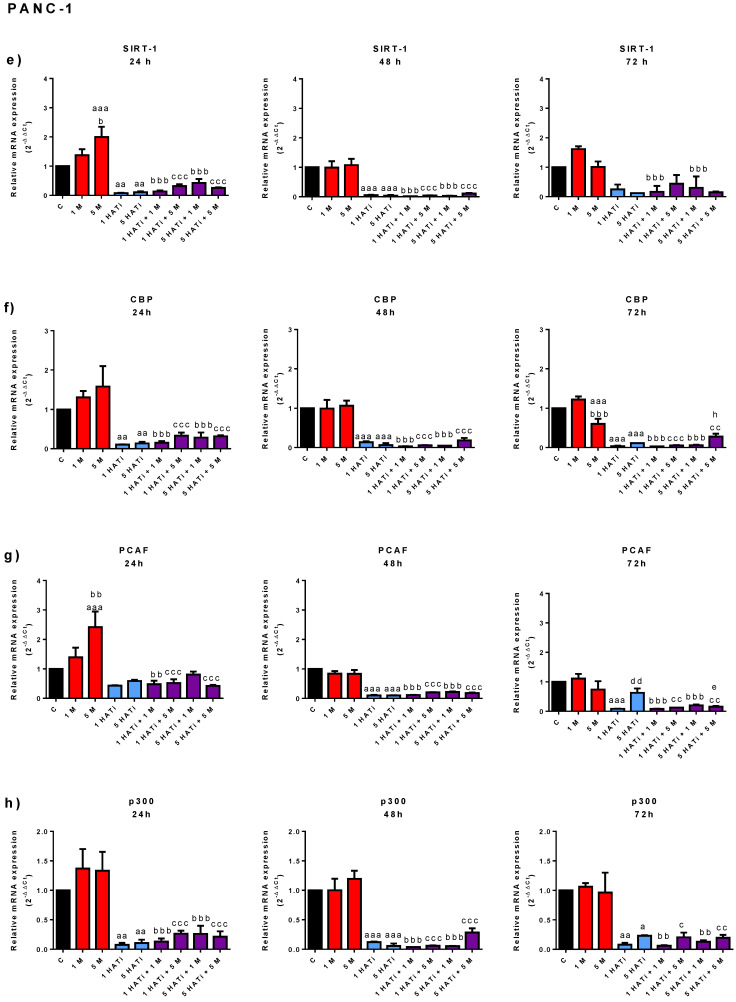
The effect of metformin, HATi, and HATi with metformin on mRNA expression of HDAC (SIRT-1) and HATs (PCAF; p300; and CBP) in 1.2B4 (**a**–**d**) and PANC-1 (**e**–**h**) cells. mRNA expression in 1.2B4 (**a**–**d**) and PANC-1 (**e**–**h**) cells was determined by qRT-PCR. The cells were treated with metformin (1 and 5 mM; red bars), HATi (1 and 5 µM; blue bars), and combination of HATi (1 and 5 µM) with metformin (1 and 5 mM; violet bars) for 24, 48, and 72 h. Data are expressed as the mean ± SD of fold change from three independent experiments in relation to the untreated control, using GAPDH as a reference gene. ^a^ *p* < 0.05; ^aa^ *p* < 0.01; ^aaa^ *p* < 0.001 vs. C; ^b^ *p* < 0.05; ^bb^ *p* < 0.01; ^bbb^ *p* < 0.001 vs. 1 M; ^c^ *p* < 0.05; ^cc^ *p* < 0.01; ^ccc^ *p* < 0.001 vs. 5 M; ^dd^ *p* < 0.01; ^e^ *p* < 0.05; ^f^ *p* < 0.05; ^h^ *p* < 0.05.

To assess the effect of metformin on HDAC (SIRT-1) and HAT (PCAF; p300; and CBP) protein expression, a Western blot analysis was performed (Figure 4). 

In 1.2B4 cells, the 24-, 48-, and 72-h metformin treatment evoked a statistically significant, dose-dependent decrease in PCAF protein expression; however, no significant effects were observed for SIRT-1, CBP, and p300. Interestingly, the 5 mM metformin treatment resulted in increased SIRT-1 protein expression after 48 h, but the expression decreased after 24 h. CBP expression decreased after treatment with 1 mM of metformin for 48 h. An insignificant rise in p300 protein expression was noted after a 48-h exposure to metformin in a dose-dependent manner.

In PANC-1 cells, the metformin treatment evoked the significant downregulation of the PCAF protein in a dose-dependent manner; however, the treatment resulted in only insignificant changes in SIRT-1, CBP, and p300 protein expression. SIRT-1 protein expression slightly increased after 24 and 48 h of exposure, but decreased in response to the 1 mM of metformin after 72 h. CBP protein expression seemed to be unchanged after exposure to 1 mM and 5 mM of metformin for 24, 48, and 72 h. The obtained results suggest that p300 protein expression slightly increased after 24 and 72 h of exposure, but not after 48 h; the changes were found to be dose dependent.

### 2.6. Metformin Acts via HAT to Induce Apoptosis of Human PC Cells

As metformin-induced apoptotic PC death appears to be associated with a pronounced reduction in the protein expression of PCAF and slight changes in p300 and CBP, the next part of the study examined the role of PCAF in the metformin-induced apoptosis of PC cells based on the use of CTK7A (an HATi) and its combination with metformin. CTK7A (hydrazinobenzoylcurcumin) is a histone acetyltransferase inhibitor of p300/CBP and PCAF histone acetyltransferases and presents minimal activity directed at other histone-modifying enzymes, such as G9a, CARM1, HDAC1, SIRT2, and TIP60. CTK7A suppresses the autoacetylation of p300 and PCAF, as well as histone acetylation. Although PCAF and CBP/p300 have been identified in the same complexes, their patterns of substrate specificity are different: CBP/p300 acetylates lysines on all four histones, whereas PCAF only acetylates lysine 14 on histone H3 [22,23].

The effect of an HATi (0.5–100 µM) and the combination of an HATi (0.5, 0.75, 1, and 5 µM) with metformin (1 and 5 mM) on the viability of PC cells was determined using MTT assays (Figure 1b,c,e,f). The HATi markedly decreased the 1.2B4 cell viability in a dose- and time-dependent manner (IC_50_ = 98.82 µM for 48 h vs. IC_50_ = 82.52 µM for 72 h), and lowered PANC-1 cell viability (IC_50_ = 99.36 µM for 24 h vs. IC_50_ = 93.44 µM for 48 h vs. IC_50_ = 87.69 µM for 72 h). Further investigations found that 1 and 5 mM of metformin and 1 and 5 µM of the HATi decreased PC cell viability by about 20%. The combination of the HATi with metformin decreased both 1.2B4 and PANC-1 cell viability by about 10%. Hence, the effects were similar to those obtained after exposure to 1 µM and 5 µM of the HATi. In PC cells, the combined treatment with metformin and an HATi (Figure 1c,f) resulted in lower viability loss in comparison to metformin alone (Figure 1a,d). Thus, these observations suggest that PCAF is involved in the metformin-induced death of 1.2B4 and PANC-1 cells.

Secondly, the effect of the HATi (1 and 5 µM) and the combination of the HATi with metformin (1, 5, and 10 mM) on the early apoptosis of 1.2B4 and PANC-1 cells was evaluated by using an Annexin V-Cy3 Apoptosis Detection Cytofluorimetric Assay Kit. The HATi treatment increased the percentage of early apoptotic 1.2B4 and PANC-1 cells in a dose-dependent manner after 24 and 48 h. These results are in line with the loss of viability detected by the MTT assay. After the 72-h HATi treatment, no early apoptotic effect was noted (Figure 2; red bars), but the percentage of dead 1.2B4 and PANC-1 cells was markedly increased (Figure 2; black bars). No significant differences in early apoptotic cell content were observed between the combination of the HATi with metformin and the HATi alone, although slightly lower values were noted for the former. These observations suggest that the HATi inhibited the apoptosis of PC cells induced by metformin. These findings are more visible when comparing the percentages of early apoptotic cells after the treatment of the HATi combined with metformin to metformin alone. The HATi appears to have a protective effect against metformin-induced apoptosis in 1.2B4 cells after 24, 48, and 72 h. Similarly, the combined treatment decreased the early apoptosis induced by metformin in PANC-1 cells after 24 and 48 h. In addition, a 72-h exposure to the combination of the HATi with metformin or the HATi alone was associated with a small decrease in the percentage of dead cells, but not to those in early apoptosis, suggesting that the response to the HATi may depend on PC cell type and exposure time.

Thirdly, to confirm whether PCAF is involved in the metformin-induced apoptosis of PC cells, the level of caspase-9 activity was measured after exposure to the HATi, metformin, and the combination of the HATi with metformin (Figure 3). Caspase-9 activity did not appear to be altered after treatment with the HATi compared to untreated cells after 24, 48, and 72 h for both 1.2B4 and PANC-1 cells. These observations are in line with results obtained via MTT and flow cytometry. However, in 1.2B4 cells, after 24 h, caspase-9 activity was markedly decreased by the combination of 5 µM of the HATi with 5 mM of metformin compared to treatment with 5 mM of metformin alone, but it was significantly increased by the combination of 5 µM of the HATi with 1 mM of metformin compared to treatment with 5 µM of the HATi alone. In addition, 1 and 5 µM of the HATi, both combined with 10 mM of metformin, significantly reduced caspase-9 activity as compared to the 10 mM of metformin used alone for 48 and 72 h. Caspase-9 activity also decreased following treatment with 1 or 5 µM of the HATi combined with 5 mM of metformin as compared to 5 mM of metformin alone after 72 h. No statistically significant changes in caspase-9 activity were noted in response to combinations of the HATi and metformin in PANC-1 cells.

The mRNA expression of PCAF, CBP, p300, and SIRT-1 after exposure to the HATi alone and the combination of the HATi with metformin was also determined (Figure 4). It was found that the response to the HATi differed between PANC-1 and 1.2B4 cells (Figure 4a–d vs. Figure 4e–h). In PANC-1 cells, all studied genes were found to be downregulated after the 24-, 48-, and 72-h treatment with the HATi. In 1.2B4 cells, the 24- and 72-h HATi treatment upregulated all the genes, whereas the 48-h treatment downregulated them. 

A cell-specific response was noted regarding the responses to the HATi alone and metformin alone. In PANC-1 cells, all genes were downregulated by both treatments, irrespective of treatment time, whereas in 1.2B4 cells, upregulation was noted after 24 and 72 h and downregulation after 48 h. 

In PANC-1 cells, no significant changes in mRNA expression were noted after exposure to the combination of the HATi with metformin compared to the HATi alone, irrespective of incubation time. The values were similar to those measured after HATi exposure, but significantly lower compared to those exposed to metformin alone. This may suggest that this cell line is more susceptible to the HATi than 1.2B4 cells. 

In addition, in 1.2B4 cells, the combined HATi and metformin treatment had different effects on each gene and incubation time. The expression of SIRT-1 mRNA seemed to be downregulated in comparison to that from the HATi treatment, but was upregulated in comparison to that of the metformin treatment after 24 and 72 h. However, the 48-h HATi and metformin treatment downregulated SIRT-1 mRNA in relation to both metformin alone and the HATi alone. PCAF mRNA expression was unaffected after 24 and 72 h of incubation with the HATi and metformin as compared to exposure to the HATi alone. PCAF mRNA expression was slightly decreased after the 48-h incubation with the HATi and metformin compared to exposure to the HATi alone, and markedly decreased compared to metformin alone. Interestingly, PCAF mRNA was significantly upregulated for the HATi and metformin compared to metformin after 24 h of incubation, but was downregulated after 72 h. 

In 1.2B4 cells, following exposure for 24 h, the CBP mRNA level was significantly higher in cells treated with the combination of the HATi with metformin compared to those exposed to metformin alone; however, only a small difference was observed between those treated with the combination of the HATi with metformin and the HATi alone. After 48 h of incubation, CBP mRNA expression was downregulated for the combination of the HATi with metformin compared to the HATi alone or metformin alone. In turn, after 72 h, CBP mRNA expression was upregulated for the HATi and metformin compared to the HATi alone and metformin alone. 

In 1.2B4 cells, after the 24-h treatment, p300 mRNA expression for the combination of the HATi and metformin was lower than the HATi alone, but higher than metformin alone. After 48 h of incubation, the level was lower for the combination of the HATi and metformin compared to the HATi alone, and significantly diminished compared to metformin alone, which was similar to the results for SIRT-1, PCAF, and CBP. In turn, after 72 h, the level was lower for the combination of the HATi and metformin compared to the HATi alone, and slightly higher compared to metformin alone. 

Hence, it appears that the mRNA expression of studied genes in 1.2B4 cells is also affected by the HATi. This may indicate that PCAF is also involved in metformin-induced apoptotic death in this cell line.

**Figure 5:**
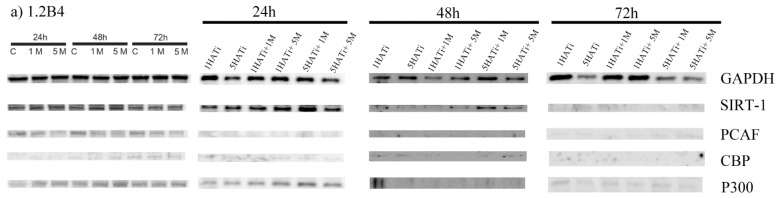

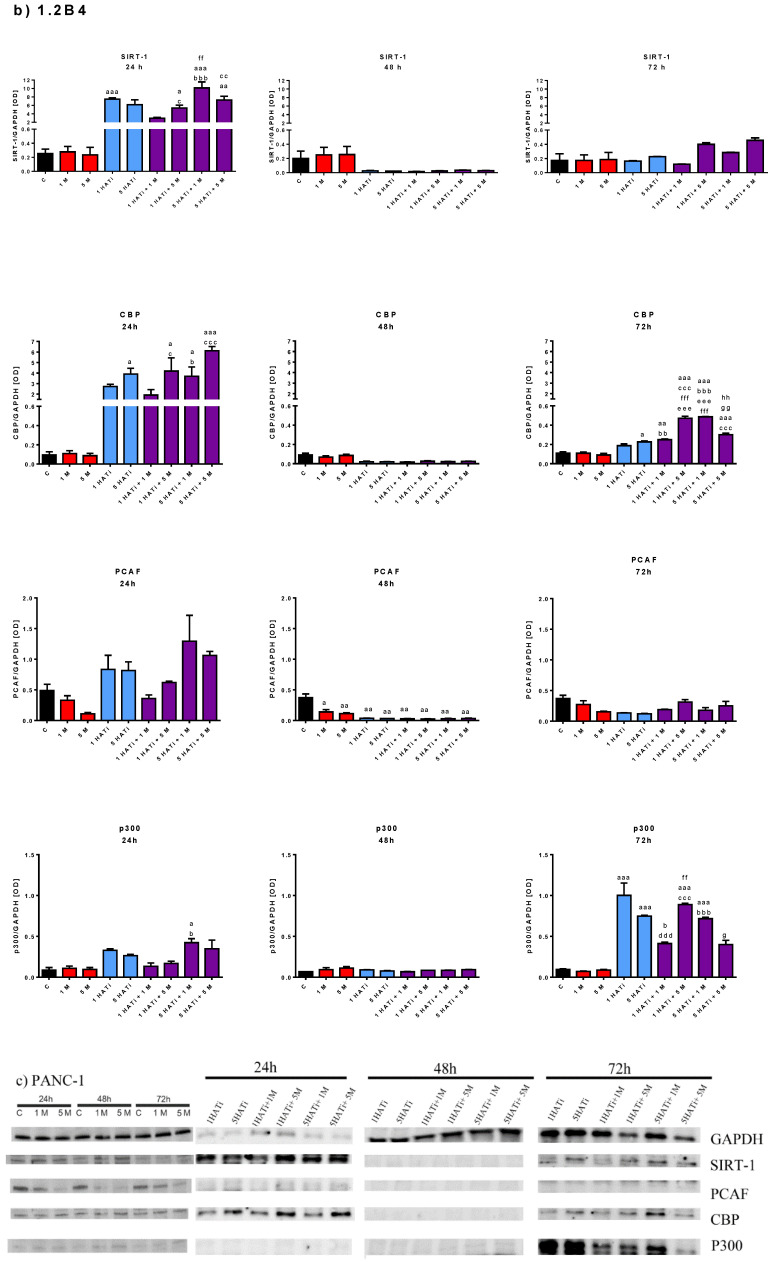

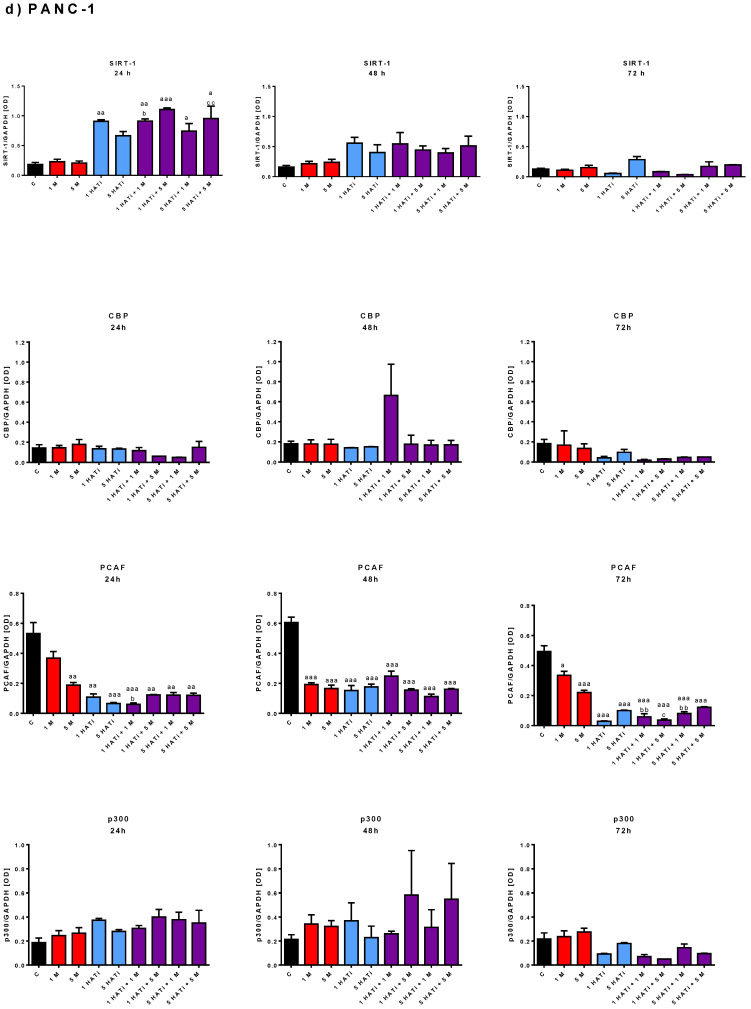
The effect of metformin, HATi, and combination of HATi with metformin on protein expression of HDAC (SIRT-1) and HATs (PCAF; p300; and CBP) in 1.2B4 (**a**,**b**) and PANC-1 (**c**,**d**) cells. Protein expression in 1.2B4 ((**a**) representative blots and (**b**) quantitative graphs) and PANC-1 ((**c**) representative blots and (**d**) quantitative graphs) cells was evaluated by Western blotting method. The cells were treated with metformin (1 and 5 mM; red bars), HATi (1 and 5 µM; blue bars), and combination of HATi (1 and 5 µM) with metformin (1 and 5 mM; violet bars) for 24, 48, and 72 h. The results are expressed as a percentage of optical density (OD) over the background ratio of target protein expression to GAPDH. Data are presented as OD means ± SEM. ^a^ *p* < 0.05; ^aa^ *p* < 0.01; ^aaa^ *p* < 0.001 vs. C; ^b^ *p* < 0.05; ^bb^ *p* < 0.01; ^bbb^ *p* < 0.001 vs. 1 M; ^c^ *p* < 0.05; ^cc^ *p* < 0.01; ^ccc^ *p* < 0.001 vs. 5 M; ^ddd^ *p* < 0.001 vs. 1 HATi; ^eee^ *p* < 0.001 vs. 5 HATi; ^ff^ *p* < 0.01; ^fff^ *p* < 0.001 vs. 1 HATi + 1 M; ^g^ *p* < 0.05; ^gg^ *p* < 0.01; ^hh^ *p* < 0.01.

Finally, the protein expression of PCAF, CBP, p300, and SIRT-1 after exposure to the HATi and the combination of the HATi with metformin was also assessed (Figure 5). As noted for mRNA expression, PANC-1 and 1.2B4 cells demonstrated different levels of protein expression in response to the HATi (Figure 5a,c). In 1.2B4 cells, after the 24-h treatment, all studied proteins were upregulated by the HATi and by the combination of the HATi with metformin as compared to metformin alone. Conversely, after a 48-h exposure, SIRT-1, CBP, and PCAF protein levels were lowered after treatment with the HATi and the combination of the HATi with metformin compared to metformin alone; no difference was observed for p300. In turn, after the 72-h treatment, SIRT-1 and PCAF levels did not differ between the HATi alone and the combination of the HATi with metformin; however, CBP and p300 were markedly elevated.

In most cases, no significant changes in protein level were found between the combination of the HATi with metformin and the HATi alone after 24, 48, and 72 h. However, the CBP protein level was significantly increased after 24 and 72 h of exposure to the combination of the HATi with metformin in comparison to the HATi alone. In turn, after a 24-h exposure, SIRT-1, CBP, PCAF, and p300 were higher following the HATi treatment compared to metformin treatment, whereas after the 72-h exposure, only CBP and p300 levels were elevated. Interestingly, after 48-h exposure, the levels of SIRT-1, CBP and PCAF were decreased for the HATi alone compared to metformin alone; however, the p300 level was unaltered.

Metformin reduced PCAF protein levels, but did not affect CBP and p300 levels in 1.2B4 cells after the 24-, 48-, or 72-h treatment. In contrast, the HATi increased CBP and p300 levels after 24 and 72 h and PCAF levels after 24 h. However, the combination of the HATi and metformin increased CBP, PCAF, and p300 levels after 24 h as compared to metformin alone. The combination of metformin and the HATi increased the rate of apoptosis, which was accompanied not only by an increase in PCAF, but also in CBP and p300. Although metformin reduced only PCAF, the HATi with metformin affected the level of all HATs and was associated with a reduction in the percentage of apoptotic 1.2B4 cells.

In PANC-1 cells, after 24 h of exposure, SIRT-1 was upregulated and PCAF downregulated due to the combination of the HATi with metformin and the HATi alone as compared to metformin alone; however, CBP and p300 were unchanged. In turn, after the 48-h incubation, PCAF was decreased due to both the HATi alone and the combination of the HATi with metformin as compared to metformin alone; SIRT-1, CBP, and p300 were unchanged. After the 72-h treatment, PCAF was significantly decreased and p300 slightly reduced for the HATi alone and the combination of the HATi with metformin as compared to metformin; SIRT-1 and CBP were unchanged. No significant changes in the studied proteins were found between the combination of the HATi with metformin and the HATi alone after 24, 48, or 72 h. Interestingly, SIRT-1 protein expression was decreased in response to the HATi and the combination of the HATi with metformin in a time-dependent manner. In turn, the p300 protein level was reduced after the 72-h exposure to the HATi alone and the combination of the HATi with metformin as compared to 24 and 48 h of exposure. The PCAF protein was also downregulated after 24, 48, and 72 h of exposure to the HATi alone and the combination of the HATi with metformin as compared to the control; however, its expression was the lowest after the 72-h incubation with the HATi and the combination of the HATi with metformin. Treatment with the HATi alone after 24 and 48 h resulted in a slightly increased SIRT-1 protein level as compared to treatment with metformin alone, and an increase in the PCAF protein level after 24, 48, and 72 h.

By contrast, in PANC-1 cells, treatment with metformin alone lowered the PCAF protein level. The HATi did not affect CBP and p300, but reduced PCAF. Metformin and the HATi did not change the level of CBP and p300, but decreased the level of PCAF in relation to metformin. Therefore, it seems that the proapoptotic effect of metformin observed in PANC-1 is associated with PCAF.

In summarizing the results of the Western blot analysis, metformin pronouncedly reduced the expression of the PCAF protein in both tested PC cell lines. PCAF protein levels corresponded to a reduction in viability, the percentage of apoptotic cells, and caspase 9 activity. Interestingly, the changes in PCAF protein levels after exposure to metformin did not reflect those in mRNA PCAF levels, which may suggest the involvement of other PCAF regulatory mechanisms in PC cells. Our results indicate that cross-talk between SIRT-1, CBP, PCAF, and p300 was involved in the apoptotic death process of 1.2B4 and PANC-1 cells. Our findings indicate that SIRT-1, CBP, PCAF, and p300 regulate each other’s activity, and that PCAF plays a key role in this relationship during the metformin-mediated apoptotic death of PC cells.

Therefore, in PANC-1 cells, the proapoptotic effect of metformin appears to be caused by PCAF, as the HATi does not significantly affect CBP or p300 levels. In turn, the proapoptotic effect of metformin found in 1.2B4 cells seems to be associated with PCAF, but the HATi also affects the levels of CBP and p300, which also suggests the involvement of CBP and p300. Thus, the origin of PC cells may determine their response to metformin connected with particular HAT involvement.

## 3. Discussion

Pancreatic cancer (PC) is the seventh leading cause of cancer death worldwide. PC can be divided into adenocarcinomas and neuroendocrine tumors (PanNETs). About 85% of PC cases are adenocarcinomas, which develop in the pancreatic ductal epithelium of exocrine glands. In turn, PanNETs are less common (<5%) tumors that develop in the endocrine tissue of the pancreas [26]. 

The present study uses PANC-1 cells to represent pancreatic adenocarcinomas, and 1.2B4 cells as both adenocarcinoma (derived from ascitic fluid) and endocrine tumor cell representatives. The pathogenesis of PC is still poorly understood, especially in relation to the involvement of enzymes responsible for the acetylation/deacetylation of chromatin. In turn, it is well accepted that the risk of PC development is lowered among T2DM patients treated with metformin. Multiple epidemiological studies have reported that patients treated with metformin have a reduced risk of cancer incidence and mortality, including ovarian [27], prostate [28], colorectal [29], and pancreas [4] cancers. Therefore, this study evaluates whether an HDAC (SIRT-1) and HATs (PCAF; p300; and CBP) are involved in the induction of apoptosis in PC cells that is evoked by metformin. It is established that epigenetic processes play a role in PC pathogenesis [30]. However, the effect of metformin on the activity of numerous epigenetic modifying enzymes is not fully understood [31].

Accumulating evidence suggests that metformin has anticancer properties [29,32]. Our findings indicate that metformin increases apoptotic cell death and caspase-9 activity in both 1.2B4 and PANC-1 cells. These results are in line with previous observations showing that metformin induces the death of various cancer cells [33,34,35,36,37,38,39,40], including cervical, ovarian, gastric, colorectal, and breast cancers, as well as melanoma. 

A study based on a flow cytometry analysis also indicates that 30 mM of metformin induced the apoptosis of ASPC-1, BxPc-3, PANC-1, and SW1990 cells, and that the proapoptotic mechanism of metformin appears to be associated with the activation of caspase-8 and -9 [41]. Interestingly, PANC-1, Panc28, and L3.6Pl demonstrated cell growth inhibition and apoptosis, and reduced Sp1, Sp3, and Sp4 expression after metformin exposure. It was also suggested that Sp downregulation triggers the anticancer activity of metformin because Sp knockdown suppresses growth and induces apoptosis in PC cells [42]. Studies conducted by Tanaka et al. indicate that metformin activated the expression of death receptor 5 (DR5), a receptor for TRAIL, and Bim with a proapoptotic function in the downstream of the TRAIL-DR5 pathway in PC cells; the authors suggest that the increased expression of these proteins may lead to the sensitization of TRAIL-induced apoptosis in PC cells [43]. Zhao et al. proposed that metformin decreases proliferation and increases apoptosis in human PANC-1 by modulating the mTOR signaling pathway [44]. In turn, Chen et al. reported that metformin suppresses cancer initiation and progression in genetic mouse models of PC [45].

The present study investigates a possible mechanism by which metformin may induce the apoptosis of PC cells, namely, its effect on enzymes targeting the acetylation status of histones. Our findings suggest that SIRT-1, PCAF, p300, and CBP may be involved in the metformin-mediated apoptosis of PC cells. Data from the literature suggest that proteins engaged in the epigenetic modification status, i.e., p300, CBP, SIRT-1, and PCAF, may be potential research and therapeutic targets in PC [46,47,48,49]. Since metformin inhibits hepatic gluconeogenesis and increases glucose uptake, it helps reduce the accumulation of glycolysis intermediates, such as pyruvate, which are then converted into acetyl-CoA, thus inhibiting the tricarboxylic acid (TCA) cycle. Under normal conditions, the citrate produced in the TCA cycle is converted to acetyl-CoA, which is required for the acetylation of histones via HATs. Additionally, the metformin-mediated activation of AMPK is associated with an increase in acetylation via the inhibition of acetyl-CoA carboxylase and the accumulation of acetyl-CoA in the cell [50]. Furthermore, the metformin-mediated activation of AMPK enhances the expression of NAMPT, which is the enzyme responsible for the first stage of NAD^+^ biosynthesis and which positively regulates the activity of NAD^+^-dependent HDAC, i.e., SIRT-1 [51].

Research on PC cells, including PANC-1, have shown that silencing SIRT-1 expression inhibits their proliferation, induces senescence and apoptosis, reduces their invasiveness, and increases their chemosensitivity [52,53,54,55,56]. Zhang et al. reported that metformin, like resveratrol, which is the best-known positive regulator of SIRT-1, promotes the increase in AMPK expression in endothelial cells through the activation of AMPK. This, in turn, causes p53 deacetylation and a decrease in its activity, thus reducing the activity of p21, which is responsible for the induction of cellular senescence [57]. However, some studies indicate that metformin can both stimulate and inhibit SIRT-1 expression in tumors, indicating that SIRT-1 may act as a tumor suppressor or oncogene depending on the type of tumor [58]. However, in the present study, metformin did not appear to have any effect on the SIRT-1 protein level in either 1.2B4 or PANC-1 cells. Studies on liver cancer cells, HepG2, showed that low metformin concentrations inhibited SIRT-1 deacetylase activity, leading to an increase in p53 acetylation and senescence [59].

No significant changes in the CBP or p300 expression were noted in 1.2B4 cells. In the case of PANC-1 cells, only CBP was downregulated in response to 5 mM of metformin after 72 h of exposure. He et al. reported that metformin induced the Ser436 phosphorylation of CBP, thus preventing the formation of the CREB–CBP–TORC2 transcription complex in hepatic cancer cells [60]. Similarly, the metformin-induced activation of AMPK in endothelial cells causes p300 phosphorylation in Ser89, reducing its enzymatic activity [61]. AMPK-dependent phosphorylation has been found to reduce the interactions between p300 and nuclear receptors, including PPARγ, and, consequently, reduces the transcription of p300-targeted genes [62,63]. Additionally, p300 and CBP have been shown to modify p53 on Lys373, 382, and 164 in the DNA-binding domain. Acetylation via p300/CBP stimulates the binding of high-affinity DNA to p53 and protects it from the negative effects of its main inhibitor: MDM2 [64].

In both cell lines, the HATi appears to act on mRNA and protein levels. In PANC-1, the HATi blocked mRNA expression of all studied genes, suggesting that they are closely related to each other. On the other hand, in 1.2B4 cells, the HATi had a different influence on individual genes and incubation times, which may suggest the participation of other acetylases and deacetylases, such as p300 and CBP, in metformin-induced apoptosis. The HATi also changed the expression of the studied proteins. Notably, some results of mRNA expression were not confirmed on the protein level; however, mRNA and protein expression are time-resolved processes, and thus, differences are to be expected between the mRNA and protein levels.

No previous data defining the role of PCAF in metformin-mediated apoptosis of PC cells could be found at the time of writing. However, in the present study, metformin was found to have a negative impact on the protein levels of PCAF in both tested cell lines. Although metformin did not appear to influence mRNA levels, previous studies indicate that PCAF is involved in the apoptosis of cancer cells. Zheng et al. reported that PCAF promotes apoptosis of hepatocellular carcinoma cells via the acetylation of histone H4 and the inactivation of Akt signaling [65]. In turn, Gai et al. note that PCAF can induce the apoptosis of hepatocellular carcinoma cells via the modulation of the GLI1/Bcl-2/BAX axis, which inhibits hepatocellular cancer progression [66]. Downregulation of both PCAF mRNA and protein was observed in gastric cancer and proposed to be correlated with poor survival. PCAF has also been suggested as a suppressor of gastric cancer via a novel PCAF–p16–CDK4 axis [67]. PCAF also acetylates transcriptional factor HOXB9, leading to the suppression of lung adenocarcinoma progression via the targeting of oncogenic protein JMJD6 [68]. 

In contrast, Malatesta et al. proposed that PCAF may have antiapoptotic properties and that PCAF downregulation triggers a reduction in the proliferation and increase in the apoptosis of medulloblastoma and glioblastoma cells. PCAF was identified as a positive cofactor of the Hh-Gli signaling pathway and cancer cell proliferation [69]. In turn, Zhang et al. indicated that the PCAF-mediated acetylation of Akt1 plays a key role in the proliferation of human glioblastoma cells [70]. 

However, little is known about the role of PCAF in the apoptosis of PC cells. Recently, Yu-Hong et al. proposed that Ras ERK1/2 promotes PC cell movement via the downregulation of H3K9ac because of MDM2-mediated PCAF degradation [49]. Our results suggest that the metformin-mediated downregulation of PCAF is related to the proapoptotic properties of metformin. It is known that PCAF, a histone acetyltransferase, is involved in global- and local-specific histone acetylation and plays a role in oncogene-mediated gene transcription, participating in the cell cycle progression of various cell types [71,72,73]. The protein was found to be related to chemical resistance, as it promotes cell growth, and the invasiveness of cancer cells. PCAF has been shown to acetylate histone 3 at 14 lysine residues, and acts as a component of large multiprotein complexes that have further histone modification abilities [74,75]. Until now, the molecular mechanism by which metformin downregulates PCAF protein expression has not yet been clarified. It could be associated with several mechanisms. Firstly, PCAF is a direct target of several microRNAs (miRNAs), such as miR-181a/b, miR-17, miR-32, miR-106b, and miR-25, which use a plethora of mechanisms to affect PCAF protein levels, e.g., they trigger its degradation or decrease its translational rate in both cancer and normal cells [76,77,78]. Our studies did not find any metformin-regulated microRNAs for which PCAF is a direct target. 

PCAF levels could also be lowered by other noncoding RNAs and long noncoding RNAs (lncRNAs) via the regulation of the rate of mRNA translation or through the induction of protein degradation by recruiting ubiquitin/proteasome components; however, this remains to be confirmed [79,80]. The effect of metformin on those lncRNAs is also not known. PCAF mRNA could be also subjected to numerous post-transcriptional modifications, e.g., m6a methylation, leading to a reduction in protein levels [81]. 

Regarding other post-translational modifications, PCAF auto-acetylation has also been shown to not only increase its activity, but also improve its stability [82]. PCAF protein synthesis and degradation could also be regulated by metformin-induced ROS and oxidative stress [37,83]. Metformin may also vastly affect the stability of PCAF by modulating its protein half-life, as was reported earlier for ΔNp63α [84]. Moreover, PCAF is a regulator of p53 signaling, as it is also subjected to MDM2-dependent control via ubiquitination and subsequent degradation [85]. Clearly, further research is needed to elucidate the mechanistic details of PCAF downregulation following metformin treatment in PC cells.

The HATi decreases the expression of cyclin E and binds to Ca^2+^/calmodulin with a high affinity, antagonizing its functions in HCT15 cells. Numerous studies have revealed Ca^2+^ and calmodulin as regulators of the cell cycle and microtubule dynamics. Ca^2+^/calmodulin-dependent protein kinase I (CaMKI) is engaged in cell progression via G1, whereas CaMKII controls G2/M and the metaphase–anaphase transition. Moreover, CaMKII may be involved in S-phase progression; however, its main role is still not fully known [86]. 

In turn, cyclin E is a key molecule for progression via the G1 phase of the cell cycle and activation of DNA replication via the interaction with and activation of cyclin-dependent kinase 2 (Cdk2), which is its catalytic partner. The regulation of the expression of cyclin E on both the ubiquitin-mediated proteolysis and the transcriptional level shows that it plays a key role in controlling the G1- and S-phase transitions. Cyclin E is a cell cycle regulatory molecule that is frequently deregulated in numerous types of cancers, where the increased expression of native or low-molecular-weight forms of cyclin E plays a crucial role in oncogenesis [87]. Thus, CTK7A may dysregulate the cell cycle, increase the viability and proliferation of cells, and promote oncogenesis by inhibiting cyclin E and Ca^2+^/calmodulin. No cell cycle analysis was performed in the present study; as such, it is difficult to speculate about the effect of CTK7A alone or in combination with metformin on cyclin E and Ca^2+^/calmodulin in PC cells, especially since we observed cell death, but not cell proliferation. 

Our results indicate that blocking PCAF reduced the apoptosis of PC cells and reduced the activity of caspase-9. It has been found that the HATi blocks the autoacetylation of PCAF and p300, as well as histone acetylation [88]. PCAF with CBP and p300 form a complex which regulates the histone acetylation status. Indeed, our results indicate that PCAF inhibition was accompanied by the decreased expression of the studied HATs and SIRT-1. Hence, it may be possible that the PCAF-, CBP-, and p300-related acetylation of histones may be involved in regulating the expression of apoptosis-related genes, although their role in the regulation of apoptosis is still not fully known. 

Our findings suggest a cross-talk between all studied biomolecules engaged in the metformin-mediated apoptosis of PC cells. It also appears that CBP, PCAF, p300, and SIRT-1 regulate the activity of each other. They are responsible for maintaining appropriate levels of histone acetylation/deacetylation. Our results have shown that the metformin-induced apoptosis of PC cells is mainly associated with PCAF, but P300 and CBP may also be involved. This effect is especially observed for PANC-1. In turn, for 1.2B4 cells, metformin affects the other studied HATs in addition to PCAF. Therefore, it is necessary to conduct further studies to determine the role of the other studied HATs in the apoptosis of PC cells induced by metformin. It should be also noted that differences in the level of studied HATs in response to metformin may also result from the origins of the PC cell lines (types of PCs). Therefore, we propose that the PCAF protein may have a key role in the metformin-mediated apoptosis of PC cells, although this requires further investigation.

Our study has several limitations. Firstly, although PANC-1 is a well-studied cell line and is a representative of pancreatic adenocarcinoma [89], the 1.2B4 cell line, as a human hybrid cell line, represents simultaneously both adenocarcinoma (derived from ascitic fluid) and endocrine tumor cells; this could significantly affect the obtained results. Secondly, the study used CTK7A, an HAT inhibitor that is not specific to PCAF only, but also to p300 and CBP: hence, although marked changes in the PCAF protein were observed after the combined treatment with metformin and the HATi in comparison to metformin alone, it is still possible that p300 and CBP may influence the results. 

Further research on the determination of the lysine 14 of histone H3 and total histone acetylation status is needed to confirm that PCAF plays a key role in metformin-induced PC death; however, a better strategy to confirm the role of PCAF would be based on using siRNA to deplete its function.

## 4. Materials and Methods

### 4.1. Cell Lines and Treatment

The 1.2B4 cell line (a human hybrid cell line formed via the fusion of the primary culture of human pancreatic islets and the human pancreatic carcinoma cell line (HuP-T3)) was purchased from the European Collection of Authenticated Cell Cultures (ECACC, UK). PANC-1 (a pancreatic duct epithelioid carcinoma cell line) was obtained from the American Type Culture Collection (ATCC, USA). Both cell lines were grown as a monolayer in the following conditions: 100% humidity, 37 °C, and an atmosphere of 95% air and 5% CO_2_. The 1.2B4 cells were cultured in RPMI 1640 (Gibco, Life Technologies, Carlsbad, CA, USA) and supplemented with 10% fetal calf serum (Gibco, Life Technologies, Carlsbad, CA, USA). PANC-1 cells were grown in Dulbecco’s Modified Eagle’s Medium (DMEM) (Gibco, Life Technologies, Carlsbad, CA, USA) and supplemented with 10% fetal bovine serum (Gibco, Life Technologies, Carlsbad, CA, USA). Both complete growth media were also supplemented with 50 IU/mL of penicillin/streptomycin (Gibco, Life Technologies, Carlsbad, CA, USA). Cells were collected after the third to fifth passage, in the logarithmic growth phase, and used in all experiments. Trypan blue (Sigma-Aldrich, Saint Louis, MO, USA) staining was used to count living cells. Metformin and the HATi (histone acetyltransferase inhibitor VII, CTK7A) were purchased from Sigma-Aldrich (Saint Louis, MO, USA). The stock solution of metformin was prepared in PBS (Sigma-Aldrich, Saint Louis, MO, USA). The stock solution of the HATi was prepared in dimethyl sulfoxide (DMSO) (Sigma-Aldrich, Saint Louis, MO, USA). The cells were exposed to metformin, the HATi, and the combination of the HATi with metformin for 24, 48, and 72 h.

### 4.2. The Cytotoxicity of Metformin, HATi, and Combination of HATi with Metformin—MTT Assay

The cytotoxicity of metformin, the HATi, and the HATi with metformin toward the PANC-1 and 1.2B4 cells was determined by using an MTT (3-(4, 5 dimethylthiazol-2-yl)-2, 5-diphenyltetrazolium bromide (Sigma-Aldrich, Saint Louis, MO, USA)) assay. PANC-1 and 1.2B4 cells were seeded in 96-well plates at a density of 2500 cells per well. After an overnight culture to achieve the logarithmic growth phase, the metformin (0.0001–100 mM), HATi, and combination of the HATi with metformin was added to cells and cultured for 24, 48, and 72 h. After the incubation was completed, 20 µL of MTT solution (5 g/L) was added to each well for an additional 4 h. Then, the medium was removed and 100 µL of DMSO (Sigma-Aldrich, Saint Louis, MO, USA) was added to each well to dissolve the formazan crystals. The absorbance was measured at a wavelength of 570 nm with a microplate reader (Spectrostar Nano, LMG Biotech, Ortenberg, Germany). The mean value of three independent PANC-1 and 1.2B4 cell cultures was taken to calculate cell viability and was reflected as a percentage of control (%). Nontreated cells (the control group) were considered to be 100%.

### 4.3. Detection of Apoptosis—Flow Cytometry

Apoptosis triggered by metformin was determined via staining using an Annexin V-Cy3 Apoptosis Detection Kit (Sigma-Aldrich, St. Louis, MO, USA). The 1.2B4 and PANC-1 cells were trypsinized and seeded at a density of 0.5 × 10^6^ cells per well on a 6-well plate. The cells were incubated overnight to achieve the logarithmic growth phase. Subsequently, 1.2B4 and PANC-1 cells were washed with PBS and a fresh medium; then, metformin, the HATi, or the combination of the HATi with metformin was added to achieve the following concentrations: 1, 5, and 10 mM of metformin, and 1 and 5 µM of the HATi. After 24 h, 48 h, and 72 h of being cultured with metformin, the HATi or the HATi with metformin, the cells were detached with a trypsin/EDTA solution and washed with PBS twice. After centrifugation, the cells were resuspended in a flow cytometry binding buffer. Next, 10^5^ cells in a 100 µL cell suspension solution, with an Annexin V-Cy3.18 Conjugate (AnnCy3) Solution or 6-Carboxyfluorescein Diacetate (CFDA) Solution were left in the dark for 15 min at room temperature. The activation of apoptosis was analyzed on a flow cytometer (FACSCalibur, BD Biosciences, San Diego, CA, USA). Each sample was tested in triplicate and three independent experiments were carried out. Results are presented as the percentage of live (green bars), apoptotic (red bars), necrotic (yellow bars), and dead (black bars) cells in each sample.

### 4.4. Activity of Caspase-9

The effect of metformin, the HATi, and the combination of the HATi with metformin on the activity of caspase-9 (apoptosis marker) was determined by using the Caspase-9 Colorimetric Activity Assay Kit (Merck, St. Louis, MO, USA) according to the manufacturer’s protocols. Each sample was tested in triplicate and three independent experiments were carried out. Data are expressed as a percentage of control. Nontreated cells (the control group) were considered to be 100%.

### 4.5. mRNA Expression—Total RNA Isolation and qRT-PCR

The isolation of total RNA from PANC-1 and 1.2B4 cells was carried out using the ReliaPrepTM RNA Cell Miniprep System (Promega, Madison, WI, USA). The quantity and quality of isolated RNA were measured with a Nanodrop 2000 (Thermo Fisher Scientific Inc., Waltham, MA, USA). Then, the reverse transcription of 1 μg of total RNA was conducted by using the High-Capacity cDNA Reverse Transcription Kit (Thermo Fisher Scientific Inc., Waltham, MA, USA), according to the manufacturer’s protocols. Obtained cDNA was used to run the qRT-PCR using the TaqMan assays (ID: Hs00914212_m1 for p300, Hs00932878_m1 for CBP, Hs01009006_m1 for SIRT-1, and Hs00187332_m1 for PCAF), GAPDH (ID: Hs99999905_m1) as a reference gene (Life Technologies, Carlsbad, CA, USA), and the TaqMan Universal Master Mix (Life Technologies, Carlsbad, CA, USA). Each sample was studied in triplicate. The relative gene expression presented as a fold change was calculated by employing the comparative Ct (ΔΔCt) methods. The equation of relative quantity: (2^−ΔΔCt^) was used [90].

### 4.6. Protein Expression—Western Botting

The isolation of the total protein from PANC-1 and 1.2B4 cells was carried out with an RIPA lysis buffer (50 mM of Tris-HCl, 150 mM of NaCl, 1% sodium deoxycholate, 0.1% SDS, and 2 mM of EDTA) that was supplemented with a protease inhibitor cocktail (Thermo Fisher Scientific Inc., Waltham, MA, USA) according to the manufacturer’s instructions. The isolated protein concentration of each sample was determined by using the Micro BCA™ Protein Assay Kit (Life Technologies, Carlsbad, CA, USA) and analyzed via immunoblotting. Fifteen micrograms of total protein per sample was separated through electrophoresis in a denaturing polyacrylamide 4–20% NuPage gel (Invitrogen, Carlsbad, CA, USA) at 140 V and 110 mA for 60 min, and transferred to a nitrocellulose membrane using the eBlot Transfer Protein System (Genscript, Piscataway, NJ, USA). The blocked membranes (5% nonfat milk in Tris-buffered saline (TBST) buffer containing 0.1% Tween 20) were incubated with a rabbit primary anti-KAT3B/P300 antibody (ab10485), anti-KAT3A/CBP (ab2832) antibody, anti-SIRT-1 (ab32441) antibody, anti-KAT2B/PCAF (ab12188) antibody, and anti-GAPDH (ab9485) antibody (Abcam, Cambridge, UK) overnight at 4 °C. The next day, the blots were washed in TBST buffer 3 times for 15 min and incubated with anti-rabbit secondary antibodies (ab205718) (Abcam, Cambridge, UK) for 4 h at 4 °C. Next, after washing in TBST buffer, the bands were visualized with the ChemiDoc MP Imaging System (Bio-Rad, CA, USA). Image J 1.34 s software (Wayne Rasband, National Institutes of Health, Bethesda, MD, USA) was employed for the densitometric analysis of visualized protein levels. GAPDH was used as a reference protein standard.

### 4.7. Statistical Analysis

Statistical analysis was conducted using GraphPad Prism 6.0 (San Diego, CA, USA). The normality of continuous variables was confirmed, and the Student’s t-test and Mann–Whitney U-test were employed to compare the treated groups and control group. An ANOVA was used to assess the differences between three or more groups. Obtained data were expressed as the mean ± SD of three independent experiments. A *p*-value < 0.05 was considered to be statistically significant.

## 5. Conclusions

In conclusion, our results indicate that PCAF is involved in the metformin-mediated apoptosis of PC cells. Metformin appears to induce apoptotic death in both 1.2B4 and PANC-1 cells, as indicated by a rise in the activity of caspase-9 in response to treatment. In turn, treatment with the HATi and the combined treatment of the HATi with metformin lowered the number of cells in early apoptosis and the activity of caspase-9. Metformin decreased PCAF protein expression in both 1.2B4 and PANC-1 cells. In turn, it seems that PCAF, CBP, p300, and SIRT-1 influenced each other’s activity, maintaining a suitable level of histone acetylation/deacetylation. Hence, we propose that the PCAF protein may play a key role in the presented cross-talk between PCAF, CBP, p300, and SIRT-1, leading to metformin-mediated apoptosis of PC cells. The obtained results may serve as an introduction for further research focused on the role of PCAF in the regulation of apoptotic gene expression.

## Data Availability

Data is contained within the article and supplementary material.

## Supplementary Materials

The following supporting information can be downloaded at: https://www.mdpi.com/article/10.3390/ph16010115/s1, Figure S1: Original flow cytometry data.
