# Supplementary material for: Metformin Induces Apoptosis in Human Pancreatic Cancer (PC) Cells Accompanied by Changes in the Levels of Histone Acetyltransferases (Particularly, p300/CBP-Associated Factor (PCAF) Protein Levels)
Source: Pharmaceuticals (Basel). 2023 Jan 12;16(1):115. doi: 10.3390/ph16010115 (PMC9863441; doi:10.3390/ph16010115)
Supplement: Supplementary file 1 [file pharmaceuticals-16-00115-s001.zip › pharmaceuticals-1994141-supplementary.pdf]

Metformin Induces Apoptosis in Human Pancreatic Cancer (PC) Cells Accompanied by Changes in the Levels of Histone Acetyltransferases (Particularly, p300/CBP-Associated Factor (PCAF) Protein Levels)

Izabela Szymczak-Pajor [1,*], Józef Drzewoski [2], Ewa Świderska [3], Justyna Strycharz [3], Anna Gabryanczyk [1], Jacek Kasznicki [4], Marta Bogdańska [5] and Agnieszka Śliwińska [1]

[1] Department of Nucleic Acid Biochemistry, Medical University of Lodz, 251 Pomorska Str.,
92-213 Lodz, Poland
[2] Central Teaching Hospital of the Medical University of Lodz, 251 Pomorska Str., 92-213 Lodz, Poland
[3] Department of Medical Biochemistry, Medical University of Lodz, 6/8 Mazowiecka Str.,
92-215 Lodz, Poland
[4] Department of Internal Diseases, Diabetology and Clinical Pharmacology, Medical University of Lodz, 251 Pomorska Str., 92-213 Lodz, Poland
[5] Student Scientific Society of Civilization Diseases, Medical University of Lodz, 251 Pomorska Str.,
92-213 Lodz, Poland
* Correspondence: izabela.szymczak@umed.lodz.pl

Supplementary meterials

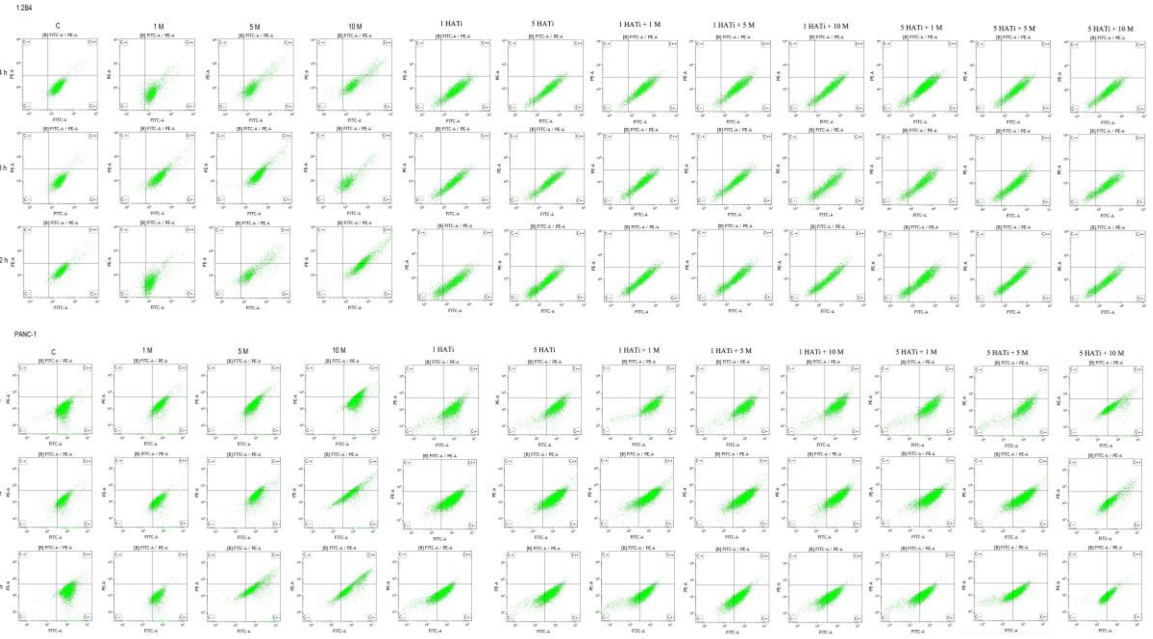

Figure S1. Original flow cytometry data.
